# The Midterm Surgical Outcome of Modified Expansive Open-Door Laminoplasty

**DOI:** 10.1155/2016/8069354

**Published:** 2016-08-03

**Authors:** Kuang-Ting Yeh, Ru-Ping Lee, Ing-Ho Chen, Tzai-Chiu Yu, Cheng-Huan Peng, Kuan-Lin Liu, Jen-Hung Wang, Wen-Tien Wu

**Affiliations:** ^1^Department of Orthopedics, Hualien Tzu Chi Hospital, Buddhist Tzu Chi Medical Foundation, Hualien 97002, Taiwan; ^2^Institute of Medical Sciences, Tzu Chi University, Hualien 97004, Taiwan; ^3^School of Medicine, Tzu Chi University, Hualien 97004, Taiwan; ^4^Department of Medical Research, Hualien Tzu Chi Hospital, Buddhist Tzu Chi Medical Foundation, Hualien 97002, Taiwan

## Abstract

Laminoplasty is a standard technique for treating patients with multilevel cervical spondylotic myelopathy. Modified expansive open-door laminoplasty (MEOLP) preserves the unilateral paraspinal musculature and nuchal ligament and prevents facet joint violation. The purpose of this study was to elucidate the midterm surgical outcomes of this less invasive technique. We retrospectively recruited 65 consecutive patients who underwent MEOLP at our institution in 2011 with at least 4 years of follow-up. Clinical conditions were evaluated by examining neck disability index, Japanese Orthopaedic Association (JOA), Nurick scale, and axial neck pain visual analog scale scores. Sagittal alignment of the cervical spine was assessed using serial lateral static and dynamic radiographs. Clinical and radiographic outcomes revealed significant recovery at the first postoperative year and still exhibited gradual improvement 1–4 years after surgery. The mean JOA recovery rate was 82.3% and 85% range of motion was observed at the final follow-up. None of the patients experienced aggravated or severe neck pain 1 year after surgery or showed complications of temporary C5 nerve palsy and lamina reclosure by the final follow-up. As a less invasive method for reducing surgical dissection by using various modifications, MEOLP yielded satisfactory midterm outcomes.

## 1. Introduction

Cervical laminoplasty is a safe and effective surgical method for treating multilevel cervical spondylotic myelopathy (MCSM) [1]. One of the most commonly used methods of laminoplasty is expansive open-door laminoplasty (EOLP) [2]. The approach, developed by Hirabayashi et al., involves fixing the opened laminae by using suture material [3]. This method was found to yield a high incidence of lamina reclosure [4]. O'Brien et al. in 1996 reported a method of applying maxillofacial miniplates and screws to provide primary resistance against lamina reclosure [5]. Between 2005 and 2011, we conducted EOLP secured by using titanium miniplates and screws for treating MCSM and observed favorable surgical results [6]. However, several predominant complications of this method were still noted; approximately 42% of the treated patients exhibited moderate to severe postoperative axial neck pain, 35% experienced a loss of range of motion (ROM), and 4.7% displayed C5 nerve palsy. To reduce the incidence rates of these complications, we developed a modified EOLP (MEOLP), which we have used since 2011 and evaluated in a retrospective study [7]. Through reducing surgical dissection by preserving the unilateral paraspinal musculature [8], preserving the C7 spinous process [9], and creating more medial gutter for reducing facet joint violation, the frequency of persistent postoperative axial neck pain and loss of ROM significantly decreased. The average length of surgical wounds after MEOLP was significantly smaller than that after conventional EOLP, and neurological outcomes for the methods were similar. Although the short-term surgical outcomes were encouraging, three major concerns remained for MEOLP at midterm follow-up. As a less invasive method, whether it can maintain adequate neurological recovery, less postoperative axial neck pain, and sufficient preserved ROM in a longer follow-up period must be clarified. Thus, the purpose of this study was to elucidate midterm (4 years) clinical and radiological results of patients with MCSM treated by MEOLP.

## 2. Material and Methods

This was a retrospective cohort study. The protocol was approved by the institutional review board of Hualien Tzu Chi Hospital, Buddhist Tzu Chi Medical Foundation, and fully informed consent was obtained from all participants (IRB103-189-B). All the patients enrolled in this study were diagnosed as having MCSM without local kyphosis of more than 15°, an anterior major lesion, or segmental instability and underwent MEOLP at Hualien Tzu Chi Hospital between March and December in 2011. Those who had a history of disorders that may have affected the baseline Japanese Orthopaedic Association (JOA) score [10], such as cerebral disorders, rheumatoid arthritis, joint disorders, and urological disorders, were excluded. The surgical procedure was a modification of unilateral open-door laminoplasty secured by using miniplates [5], which has been fully described previously [7]. Through unilateral paraspinal muscle dissection and cutting of spinous process, the bilateral laminae were approached. C7 partial laminectomy was performed at first and the border of spinal cord was exposed. We then created the bilateral gutters based on the diameter of exposed spinal cord. The gutters were often less than 0.8 cm lateral to the spinous process and just lateral to the border of spinal cord without visional exposure of the facet joints. Then C3–C6 laminae were separately elevated and fixed with titanium miniplates and screws. After checking the spinal cord free from compression, we closed the wound to finish this procedure. For the first 3 months after surgery, the patients wore hard collars and performed adequate neck extension exercise. All of them were followed up for at least 4 years. The follow-up rate of these patients was 100%.

All patients underwent follow-up examinations every 3 months for the first year after surgery and once per year thereafter. We collected the demographic data of the patients, namely, age, sex, body mass index, preexisting medical comorbidities, and smoking history. Clinical outcome data included neurological and functional status assessed by using the neck disability index (NDI) score [11], JOA score and recovery rate (100 × [final JOA score − preoperative JOA score]/[17 − preoperative JOA score]) [6], and visual analog scale (VAS) score for axial neck pain, which was defined as nuchal and/or scapular pain. Pain intensity was graded as severe (VAS 8–10), moderate (4–7), or mild (0–3), in accordance with a previous study [12]. Maximal flexion and neutral and maximal extension were examined by taking lateral radiographs of the cervical spine obtained before surgery and at regular intervals after surgery thereafter. Parameters of sagittal alignment of the cervical spine included cervical lordosis (CL) and cervical sagittal vertical axis (CSVA). CL was measured as the C2–C7 angle formed by two lines drawn parallel to the posterior margin of the vertebral body on a radiograph in the neutral position [13]. CSVA was measured as the distance between the vertical axes through the center of the C2 body and posterior border of the upper endplate of C7 [14]. The C2–C7 ROM of the cervical spine was calculated by subtracting the maximal flexion C2–C7 angle from the maximal extension C2–C7 angle [15].

Data are presented as the mean ± SD. An independent* t*-test was used to analyze the difference between the preoperative and postoperative scores. A* P* value less than 0.05 was considered statistically significant.

## 3. Results

Forty-five male and 20 female patients were enrolled in this study. The demographic data were presented in Table 1. More female patients than male patients had a history of diabetes mellitus. The female patients had a smaller mean preoperative CSVA and less favorable preoperative JOA score. The mean age of all patients at the time of surgery was 60.5 years, and the mean length of wound was 4.8 cm. The mean duration of follow-up was 48.5 months.

### 3.1. Axial Neck Pain

The mean VAS of preoperative axial neck pain was 2.9, and it decreased to 2.6 at 3 months after surgery (Table 2). The mean VAS of axial neck pain at 48 months after surgery was 1.3. Thirteen patients (20%) experienced moderate neck pain at the third postoperative month; the symptom completely decreased to mild pain at 1 year after the operation. None of the patients experienced aggravated or severe neck pain from 1 to 4 years after surgery.

### 3.2. Functional Score

The mean JOA score improved significantly from 11.0 before surgery to 15.6 at 1 year after surgery (Table 2). At the final follow-up, the mean score increased slightly to 16.3, representing a mean recovery rate of 82.3%. The mean NDI score decreased from 30.7 preoperatively to 11.5 at the 48-month follow-up. The mean Nurick score also improved from 2.7 preoperatively to 0.7 at 4 years after the operation. None of the 65 patients showed worsening of myelopathy after surgery.

### 3.3. Radiographic Parameters

**Figure 1:**
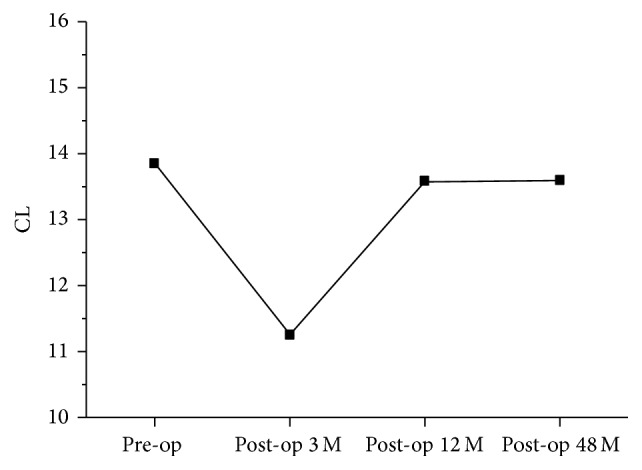
The change of C2–C7 lordotic angle (CL) from preoperative status to final follow-up at postoperative 4 years. The lowest point was at postoperative 3 months.

**Figure 2:**
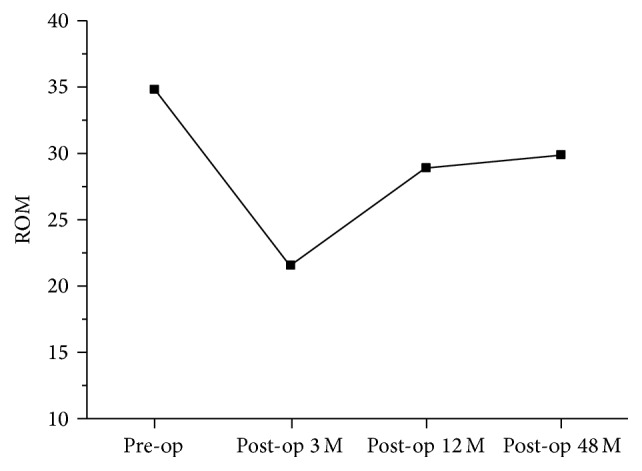
The change of C2–C7 range of motion (ROM) from preoperative status to final follow-up at postoperative 4 years. The lowest point was at postoperative 3 months.

The mean CL decreased but not significantly, declining from 13.9° preoperatively to 13.6° at 4 years after the operation.  Figure 1 shows that CL decreased to the lowest point at the third postoperative month and recovered gradually afterward. The mean CSVA increased from 19.6 mm preoperatively to 22.3 mm at 4 years after the operation (*P* < 0.05). Mean C2–C7 ROM decreased from 34.9° before surgery to 29.9° at the 48-month follow-up (*P* < 0.05).  Figure 2 shows that ROM decreased to the lowest point at the third postoperative month and gradually improved afterward. Approximately 85% ROM was preserved at 4 years after the operation. Progression of C6/7 degeneration was found in two patients (3.1%) at the final follow-up, and both patients had intermittent moderate neck pain with gradual onset of radiculopathy but near normal life quality.

### 3.4. Complications

One patient exhibited poor wound healing and received debridement and reclosure in the operation room. No patient had experienced temporary C5 nerve palsy or lamina reclosure at the final follow-up.

### 3.5. Case Report

**Figure 3:**
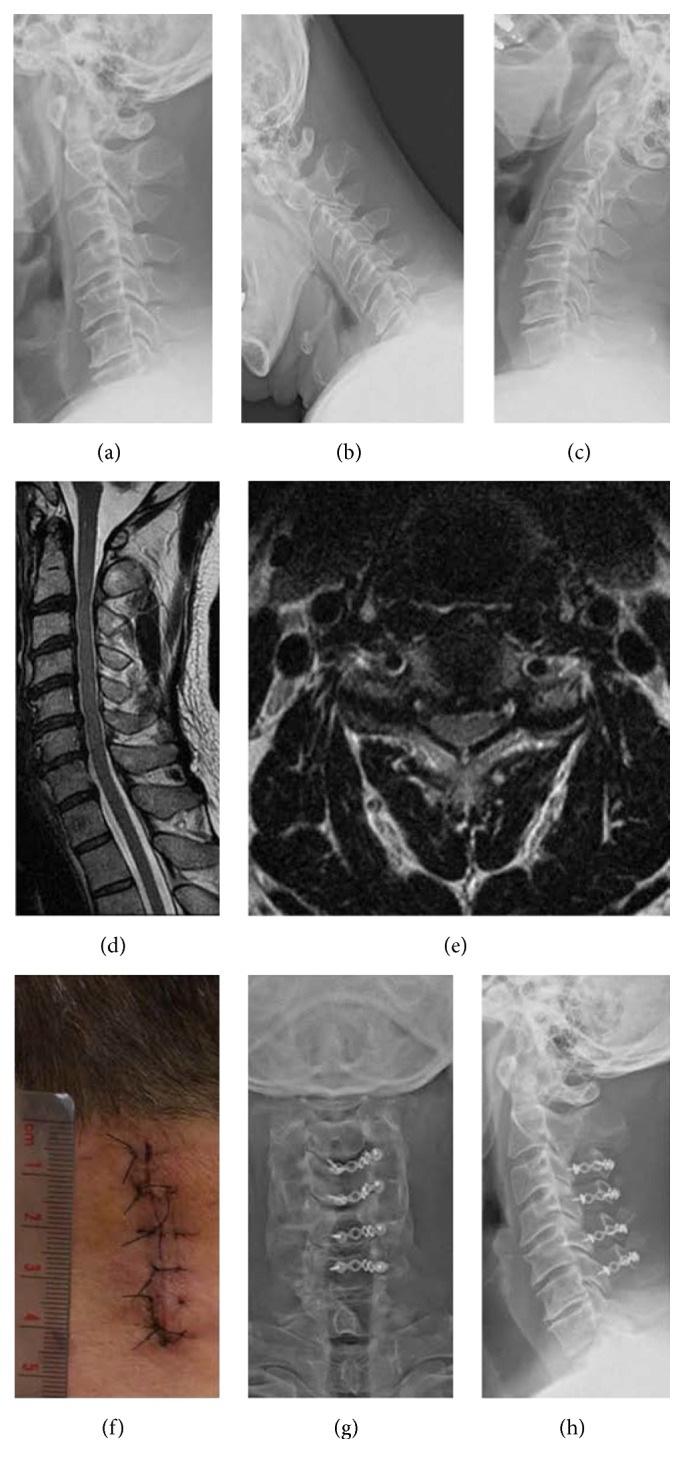
Preoperative X-ray in this case showed C3–C7 spondylosis (a) without segmental instability and local kyphotic deformity (b and c). T2 weighted MRI revealed C3–C7 stenosis at sagittal plane (d) and banana shape of the compressed spinal cord at axial plane (e). The surgical wound was about 4 cm (f). Postoperative plain films showed well alignment of C3–C6 laminoplasty and C7 partial laminectomy at anterior to posterior (g) and lateral (h) views at 1 month.

**Figure 4:**
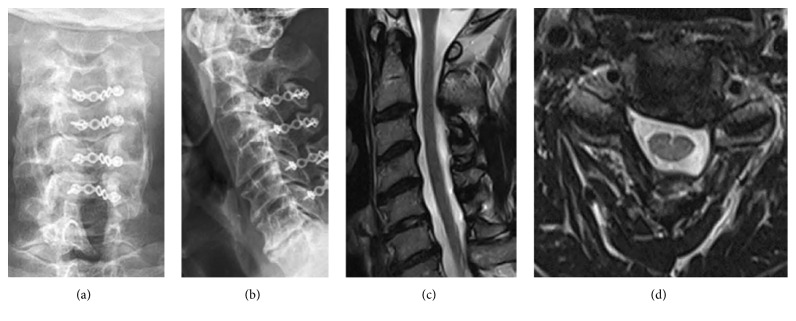
Postoperative X-ray at 4 years demonstrated well cervical curvature with C6-C7 disc space narrowing at anterior to posterior (a) and lateral (b) views. Post-op MRI revealed patent spinal cord without compression at sagittal plane (c) and axial plane (d).

A 47-year-old male teacher presented with bilateral hand clumsiness, numbness in four limbs, and impaired tandem gait. He was found to have preoperative JOA score of 11, Nurick score of 2, and preoperative neck pain VAS of 3. Plain film revealed no instability or local kyphosis (Figures 3(a), 3(b), and 3(c)). His preoperative CL was 14° and preoperative ROM was 35°. Cervical MRI showed C3–C7 stenosis with substantial compression of the spinal cord but without any anterior main budging lesion over these segments (Figures 3(d) and 3(e)). We performed MEOLP on the patient (Figures 3(f), 3(g), and 3(h)). His neck pain VAS score was 2 at 3 months, which decreased to 0 at 6 months after operation. His postoperative JOA and Nurick scores were 17 and 0, respectively, at both 12 and 48 months after surgery. At 4 years after surgery, the patient exhibited a 100% JOA recovery rate, 10° CL, and 28° ROM (Figures 4(a), 4(b), and 4(c)), with 60% ROM preserved. A postoperative MRI at 4 years after surgery revealed a patent spinal cord without compression (Figure 4(d)). The patient expressed high satisfaction with this operation and recovery.

## 4. Discussion

This study revealed favorable clinical and radiographic outcomes of MEOLP at 4 years postoperatively. We reduced the complication rates by minimizing surgical dissections of conventional EOLP [7]. Several less invasive methods, such as muscle preservation concepts of exposure of the cervical spinal laminae developed by Shiraishi [8], selective laminoplasty [16], C3–C6 laminoplasty [17], and cervical laminoplasty with C3 laminectomy [18], have been reported for preventing surgery-associated problems such as axial neck pain and loss of cervical lordosis by reducing damage to the paraspinal muscles and nuchal ligaments. Our MEOLP combines the advantages of these methods and has comparable neurologic recoveries and very less axial neck pain during the longer period of follow-up [16, 18, 19]. Compared to C3–C6 laminoplasty developed by Hosono et al., our method also restores better postoperative neck ROM and cervical lordosis at medium-term follow-up [17, 19].

The current results showed significant improvements in neck pain at 3 months, 1 year, and 4 years following surgery. None of the patients reported aggravated or severe neck pain after 1 year following surgery. Aggravated axial neck pain is one of the most common complications of EOLP, with a reported incidence of 30%–60% [20]. The main causes include the severe damage to the paraspinal muscle and nuchal ligament. One cadaveric study revealed that laminoplasty without the dissection of muscles attached to the C7 spinous process preserves the trapezius as well as the rhomboideus more effectively than do conventional methods [21]. Our MEOLP method reduces muscle damage by dissecting unilateral paraspinal muscle and sawing the spinous process to approach the other side of the laminae. The method also reduces injury to the nuchal ligament by preserving muscles attached to the C7 spinous process. Two patients reported intermittent moderate neck pain with left C7 radiculopathy at the final follow-up because of progressive C6/7 disc degenerative change. Both patients had preoperative C6/7 disc space narrowing without segmental instability or local kyphosis. Partial C7 laminectomy may aggravate this condition.

Favorable neurologic recovery and significant improvement of disability were noted in the patients at the final follow-up without deterioration. In addition, the patients did not exhibit C5 nerve palsy (a common short-term complication [22]) or lamina reclosure (a common medium- and long-term complication [23]). Laminoplasty decompresses the spinal cord through lamina elevation and secure fixation; an overly wide opening may cause facet joint violation and a higher incidence of C5 nerve traction injury [24]. Furthermore, an overly lateral approach may damage the posterior rami of the spinal nerves and cause paraspinal muscle atrophy and disability [25]. Our MEOLP method achieves lamina elevation by creating more medial bilateral gutters to approximately 7 mm from the spinous process. The distance was determined according to three findings: (1) the border of the spinal cord measured during partial C7 laminectomy, (2) the measurement of the extent of the spinal cord width in cadaveric study, and (3) the measurement of spinal cord diameters from the axial MRI view of C3–C7 in 200 patients. We found that the average distance between the facet joints was 24 mm but the average cord width was only 14 mm. The axial MRI and cadaveric research revealed that the facet joints were located so laterally from the lateral borders of the spinal cord that they were not necessary to be identified and approached while creating the gutters on the laminae. Based on the information from MRI study, cadaveric dissection, intraoperative findings, postoperative MRI work-up, and postoperative neurologic improvement, we could say that the modified EOLP could afford enough cord decompression.

Preservation of more than 80% ROM and restoration of cervical lordosis to near preoperative levels were noted at 48 months following MEOLP. This result may be attributable to repairing the semispinalis cervicis (SC) [26] and reducing facet joint violation [27]. Failure to repair the SC can cause substantial axial neck pain and loss of lordosis [28]. Preserving more musculature can not only reduce axial neck pain but also preserve and restore more neck ROM [29].

More than 80% JOA recovery rate was noted in this group of our patients who received modified laminoplasty technique. We strictly selected our patients by the indications of laminoplasty as multilevel cervical myelopathy without segmental instability, local kyphosis, or anterior major foci. We also convinced patients to receive the operation when the diagnosis of symptomatic myelopathy was confirmed so that the treatment was not delayed. The earlier the myelopathy is surgically treated, the more the neurologic functions recover. Then we followed up these patients closely and taught them to do neck extension exercise under hard collar protection aggressively. Although we had good neurologic recovery and functional outcomes in the medium-term follow-up, the long-term outcomes of the modified technique still need to be clarified under the influence of degenerative change of cervical spine.

The results of this study are limited because of the relatively small case number of MEOLP, the retrospective design, and the lack of a comparison group. In addition, longer term follow-up is required to evaluate the progressive degenerative disc change within the laminoplasty and adjacent segment [30] following MEOLP.

## 5. Conclusion

We conclude that our MEOLP method is an effective and less invasive surgical procedure for treating patients with MCSM. Furthermore, the method was found to provide satisfactory medium-term results by preserving muscles and the nuchal ligament attached to the C7 spinous processes, minimizing injury of paraspinal extensor musculature, reducing facet joint violation, and ensuring adequate lamina opening. This method provided favorable clinical outcomes with fewer complications resulting from avoiding unnecessary dissection.

## Figures and Tables

**Table 1:** Demographics (*n* = 65).

	Male	Female	Total
*N*	45	20	65
Age	60.47 ± 10.44	63.75 ± 10.66	61.48 ± 10.53
Body mass index			
Normal	21 (46.7%)	8 (40.0%)	29 (44.6%)
Underweight	0 (0.0%)	1 (5.0%)	1 (1.5%)
Overweight	20 (44.4%)	6 (30.0%)	26 (40.0%)
Obese	4 (8.9%)	5 (25.0%)	9 (13.8%)
Diabetes mellitus (%)	5 (11.1%)	7 (35.0%)	12 (18.5%)
Hypertension (%)	9 (20.0%)	8 (40.0%)	17 (26.2%)
Cardiovascular disease (%)	13 (28.9%)	5 (25.0%)	18 (27.7%)
Smoke (%)	16 (35.6%)	3 (15.0%)	19 (29.2%)
Functional score			
VAS	2.8 ± 1.9	3.0 ± 2.3	2.9 ± 2.0
NDI	30.6 ± 4.6	30.8 ± 4.8	30.7 ± 4.6
JOA score	11.3 ± 1.5	10.4 ± 1.6	11.0 ± 1.5
Nurick score	2.6 ± 0.9	2.9 ± 1.0	2.7 ± 0.9
Radiographic parameters			
CL (°)	13.0 ± 9.9	15.8 ± 8.6	13.9 ± 9.6
C2–7 SVA (mm)	22.3 ± 11.9	13.4 ± 9.4	19.6 ± 11.9
ROM (°)	34.7 ± 12.5	35.1 ± 13.4	34.9 ± 12.7

Data are presented as *n* (%) or mean ± standard deviation.

**Table 2:** Preoperative and postoperative clinical and radiographic status (*n* = 65).

Item	Pre-op	Post-op			*P* value
		3 M	12 M	48 M	
Axial neck pain					
VAS	2.9 ± 2.0	2.6 ± 2.1	1.9 ± 1.6	1.3 ± 1.0	<0.001^*∗*a^
Functional recovery					
NDI	30.7 ± 4.6	—	13.2 ± 2.2	11.5 ± 4.6	<0.001^*∗*a^
JOA score	11.0 ± 1.5	—	15.6 ± 3.4	16.3 ± 1.4	<0.001^*∗*a^
Nurick score	2.7 ± 0.9	—	1.2 ± 1.3	0.7 ± 1.0	<0.001^*∗*a^
*JOA recovery rate (%)*				*82.3 ± 16.7*	
Radiographic change					
CL (°)	13.9 ± 9.6	11.3 ± 7.8	13.6 ± 8.3	13.6 ± 8.5	0.700^a^
CSVA (mm)	19.6 ± 11.9	23.1 ± 12.8	21.8 ± 13.2	22.3 ± 13.6	0.031^*∗*a^
ROM (°)	34.9 ± 12.7	21.6 ± 8.6	29.0 ± 10.0	29.9 ± 10.7	<0.001^*∗*a^

Data are presented as mean ± standard deviation.

^a^Post-op 48 M versus pre-op.

^*∗*^ *P*  value < 0.05 was considered statistically significant after test.
